# Being targeted: How counter-arguing and message relevance mediate the effects of cultural value appeals on disease prevention attitudes and behaviors

**DOI:** 10.3389/fpsyg.2022.1018402

**Published:** 2022-09-29

**Authors:** Xiaodi Yan

**Affiliations:** Guangming School of Journalism and Communication, China University of Political Science and Law, Changping, China

**Keywords:** cultural value appeals, individualism-collectivism, counter-arguing, message relevance, infectious disease, COVID-19, health persuasion

## Abstract

This study investigated the effects of cultural value appeals in health persuasion. Situated in the COVID-19 pandemic, this study examined if and how individualistic and collectivistic appeals can improve attitudes and behaviors related to the use of face masks among European Americans and Asian Americans. Results showed that for European Americans, collectivistic vs. individualistic appeals were more effective to improve attitudes and behavioral intention. Perceived message relevance and counter-arguing were significant mediators explaining the effects. For Asian Americans, both individualistic and collectivistic appeals predicted significant changes in attitudinal and behavioral outcomes. These findings have important theoretical and practical implications.

## Introduction

Adapting to audience's values is thought to be an important element in effective persuasion. A large body of research has demonstrated that advertisements with appeals adapted to the audience's cultural values were more effective than appeals that are unadapted (Han and Shavitt, 1994; Aaker and Williams, 1998; Aaker and Schmitt, 2001; Hoeken et al., 2003; Chang, 2006). A meta-analysis found that cultural value appeals were more persuasive than unadapted ads overall (Hornikx and O'Keefe, 2009).

In the field of public health, there is also growing awareness that communication about health needs to take cultural characteristics into account to be most effective (see, e.g., Kreuter et al., 2003; Kreuter and McClure, 2004; Kreuter and Haughton, 2006; Dutta, 2007; Betsch et al., 2016). The cultural sensitivity approach (Resnicow et al., 1999) focuses on developing culturally appropriate health communication strategies. Cultural values and beliefs are to be recognized, reinforced, and built on to provide context and meaning to health information. One of the most important cultural value dimensions is individualism—collectivism, based on which health messages are often framed.

### Individualistic and collectivistic appeals

Individualism—collectivism (Hofstede, 1984; Markus and Kitayama, 1991; Triandis, 1995) as a group characteristic is “the degree to which people in a society are integrated into groups” (Hofstede, 2011, p. 11). In individualistic cultures, social ties between individuals tend to be loose, and everyone takes care of themselves. People with individualistic tendency focus on rights above duties and place priority on personal goals and desires over group goals or social welfare. In contrast, collectivistic cultures are characterized by strong social bonds and interconnectedness with in-group members. They tend to focus on social, mutual obligations and are motivated to fulfill group expectations and goals over individual rights or personal concerns (Hofstede, 1984, 2011; Markus and Kitayama, 1991; Triandis, 1995).

In health communication, persuasive messages address cultural values of individualism and collectivism through different message frames (Resnicow et al., 1999; Murray-Johnson et al., 2001; Ko and Kim, 2010; Uskul and Oyserman, 2010; Han and Jo, 2012; Lee and Park, 2012; Chung and Ahn, 2013; Yu and Shen, 2013). An individualistic appeal focuses on a person's own physical body and wellness and emphasizes the personal consequences of health-related behaviors. In contrast, a collectivistic appeal focuses on one's group and their collective health and emphasize the relational consequences of health-related behaviors. For instance, an anti-smoking campaign could use an individualistic appeal by saying that “When you smoke, you suffer…You inhale poisons…affect your heart, lungs…Take care of yourself” (Lee and Park, 2012, p. 76) or it could employ a collectivistic appeal by saying that “When you smoke, they suffer…Your friends and family breathe your smoke, inhaling poisons…affect their hearts, lungs…Take care of them” (Lee and Park, 2012, p. 76).

Individualistic-collectivistic framing is to be distinguished from individual-societal framing (Iyengar, 1990). Individual-societal framing is about how news media attribute responsibility for social problems (Iyengar, 1990; c.f., De Vreese, 2005; Kim, 2015). For instance, when news media present a social problem as a particular instance of a person (i.e., individual framing), responsibility is assigned to the individual, so the solution is to change individual's deficiencies. Alternatively, when news media frame a social problem as a general outcome (i.e., societal framing), responsibility is assigned to flawed social conditions, so it calls for changes in social forces such as government policies. Individualistic-collectivistic framing is different in that it addresses cultural value orientations—what a person thinks is important and what guides one's actions and behaviors. Appealing to one's cultural values is intended to make the persuasive arguments more effective in terms of changing individual's attitudes and behaviors.

### The effects of individualistic vs. collectivistic appeals

Although the effectiveness of culturally targeted health communication is recognized theoretically, empirical evidence is limited and mixed (Murray-Johnson et al., 2001; Ko and Kim, 2010; Uskul and Oyserman, 2010; Han and Jo, 2012; Lee and Park, 2012; Chung and Ahn, 2013; Yu and Shen, 2013). For instance, looking at cancer screening campaign ads, Han and Jo (2012) found that U.S. participants had more favorable attitudes to individualistic appeals than collectivistic appeals, whereas Japanese participants responded more favorably to collectivistic appeals than individualistic appeals. However, the same pattern was not observed for behavioral intention. Additionally, Han and Jo (2012) discovered no difference in the effects of individualistic and collectivistic appeals on attitudes or behavioral intention among Korean participants. Moreover, Yu and Shen (2013) looked at loss-framed messages about getting a flu shot. They found that among American participants, those who read an other-appeal reported more favorable attitudes and higher behavioral intention than those who read a self-appeal, contrary to what one would expect. Among Hong Kong Chinese participants, other-appeal was more effective only for behavioral intention but not for attitudes. Another example is Ko and Kim (2010) studying safe sexual practices and condom use. European Americans were found to report higher behavioral intention after being exposed to a message on relational risk than a message on personal risk, unexpectedly. Asian Americans, on the other hand, did not differ in their behavioral intention when they saw a relational-risk-framed message or a personal-risk-framed message. The status quo of existing literature on this topic calls for further investigation.

Moreover, research on culturally targeted health communication is important as the United States become increasing ethnically diverse. Different ethnic groups have maintained separate cultural identities and cultivated their own cultural value systems (Phinney, 1996; Cokley, 2007). For instance, compared to European Americans, Asian Americans have been characterized by the importance of fulfilling obligations to the in-group, placing group goals over individual goals, and maintaining social harmony (Oyserman et al., 2002; Leong et al., 2006; Schwartz et al., 2010). A meta-analysis by Oyserman et al. (2002) found that Asian Americans were lower in individualism and higher in collectivism than European Americans. As such, effective health communication to European and Asian Americans may employ different strategies. The first research question investigates the persuasive effectiveness of individualistic and collectivistic appeals for European and Asian Americans.

Research Question 1: What are the effects of individualistic vs. collectivistic appeals on attitudinal and behavioral outcomes among European and Asian Americans?

### Potential mechanisms for the effects of individualistic vs. collectivistic appeals

Furthermore, another question that remains unclear in existing literature is why we obtain the observed effects of cultural value appeals. One explanation is inspired by the Elaboration Likelihood Model (ELM; Petty and Cacioppo, 1981). Depending on the degree of elaboration, recipients can engage in two different approaches to message processing: The central route engages in a thoughtful examination of the message and careful scrutiny of the arguments contained in the message; The peripheral route, on the other hand, involves heuristic principles and peripheral cues. ELM argues that persuasive outcomes obtained through the central route of processing are likely to be more stable over time and predictive of future behaviors, compared to that through the peripheral route of processing.

ELM suggests that one of the factors that influence the degree of elaboration is motivation. When the message is relevant and recipients have high involvement, they are motivated to engage in central route of processing. *Message relevance* is when recipients perceive the message to be related or applicable to them and their situation. Cultural value appeals may be perceived as more relevant, as they respond to recipients' cultural markers and provide culturally congruent views of and solutions to the issue at hand (Kreuter and Wray, 2003; Rimer and Kreuter, 2006; Hawkins et al., 2008; Jensen et al., 2012). As such, cultural value appeals are more likely to stimulate *message engagement*, which may include close attention, effortful thinking, and careful examination of the message—characteristics of the central route of processing. Therefore, cultural value appeals may achieve persuasive outcomes through recipients' perceived message relevance and message engagement.

However, ELM also points out that there are challenges to persuasion through the central route of processing. Effortful processing may evoke *counter-arguing* that could lessen message effects. Especially when the advocated position in the message is counter-attitudinal, recipients are likely to generate negative thoughts of the message, which renders the persuasive attempt unsuccessful. The issue of psychological reactance may be more salient with cultural value appeals. When recipients' cultural values are coupled with counter-attitudinal arguments, recipients are likely to experience psychological discomfort and cognitive dissonance (Festinger, 1957). Recipients may feel their underlying values are challenged and their cultural identity is offended and threatened. To resolve these negative feelings, recipients are likely to engage in defensive processing of the message and counter-arguing to minimize the message (Ko and Kim, 2010; Yang and Nan, 2019). Therefore, cultural value appeals may fail to obtain persuasive outcomes due to recipients' counter-arguing with the message. The second research question attempts to explore these potential explanations for why cultural value appeals persuade or fail to do so.

Research Question 2: What are some mechanisms through which individualistic vs. collectivistic appeals affect attitudinal and behavioral outcomes?

Taken together, empirical evidence on the effectiveness of cultural value appeals in health communication is mixed, and investigations on the mechanisms through which cultural value appeals influence persuasive outcomes are insufficient. Therefore, the current study attends to these gaps in literature.

## Method

### Study context

The COVID-19 pandemic has had significant socio-economic impacts around the world (e.g., Nicola et al., 2020). Research showed that a large portion of the spread of COVID-19 occurs through airborne aerosols (i.e., tiny viral particles that can float in the air and remain infectious for hours) when infected individuals breathe, speak, cough, or sneeze (Anderson et al., 2020; Morawska and Cao, 2020). In October 2020, when this study was conducted, COVID-19 vaccines had not been available to the public, so universal masking is one of the most effective measures to reduce airborne transmission of COVID-19 (Brooks et al., 2020; Chu et al., 2020; Greenhalgh et al., 2020; Leung et al., 2020; Lyu and Wehby, 2020; Pleil et al., 2020; Prather et al., 2020; Verma et al., 2020).

Despite the health benefits of face masks during COVID-19, there has been great resistance to wearing masks in the United States (Beer, 2020; De Nova, 2020; Li, 2020). Survey data collected between March 2020 and January 2021 by University of Southern California (Key, 2021) showed that only about half of Americans (51%) said they mostly or always wore a mask when in close contact with people outside their household, with White Americans the least likely (46%) to consistently wear a mask while doing so. While the vast majority of Americans wore masks for grocery shopping, only about 20% wore a mask most or all of the time when they visited someone else's home. Additionally, only about half of Americans consistently wore a mask when they attended religious service (60%) or visited a bar or restaurant (53%).

Given the context, investigating ways to improve attitudes and behaviors related to the use of face masks to prevent the spread of an infectious disease was both appropriate and important.

### Participants

Participants (*N* = 418) were recruited from a paid subject pool through *Qualtrics*^xm^ Research Suite in October 2020. Among them, 205 participants self-identified as European Americans, and 213 participants self-identified as Asian Americans. Participants' age ranged from 18 to 86 years (*M* = 45, *SD* = 16.51). About half of the participants were female (50.6%). The average political orientation score is 4.16 (*SD* = 1.75) on a scale ranging from 1 (*very conservative*) to 7 (*very liberal*). More than half of the participants were married (58.0%), Christian (55.0%), and had an education level above bachelor's degree (59.6%). Participants came from 43 states across the United States, with the most from California (19.4%), New York (13.2%), Florida (6.9%), Texas (5.5%), Pennsylvania (4.2%), Illinois (4.0%), Georgia (3.2%), Ohio (3.0%), Indiana (2.7%), Michigan (2.7%), and Virginia (2.7%).

### Design and procedure

This study was a 2 (message frame: individualistic vs. collectivistic) × 2 (audience ethnicity: European vs. Asian American) between-subjects pretest-posttest design. Cell sizes were approximately equal. Attitudes and behavioral intention were the outcomes of interest. All procedures and materials were approved by institutional review board.

After obtaining informed consent, participants completed an online survey. Participants first indicated their current attitudes toward wearing masks and current behaviors of wearing masks prior to message exposure. Next, participants reported their standings on a collectivism scale. Participants also provided demographic information on their ethnicity, age, gender, political orientation, religion, marital status, education, and state of residence. Then, participants were randomly assigned to view one of the posters. The posters featured a *Mask Up* campaign and were framed as either an individualistic or collectivistic appeal. Participants viewed the poster for at least 20 s before they reported their levels of message engagement, perceived relevance of the message, and their counter-arguing while viewing the message. Finally, participants indicated their attitudes toward wearing masks and future behavioral intention to wear masks after message exposure. The lapse between the pretest and posttest measures was about 15.25 min on average.

### Stimuli

Two versions of a poster featuring a *Mask Up* campaign (see Supplementary Appendix A) were created. Images were attributed to resources from Freepik.com. Message frames were manipulated through both texts and pictures, following practices in previous research (Murray-Johnson et al., 2001; Ko and Kim, 2010; Uskul and Oyserman, 2010; Han and Jo, 2012; Lee and Park, 2012; Chung and Ahn, 2013; Yu and Shen, 2013):

To create an individualistic vs. a collectivistic appeal, messages framed wearing a mask as an individual behavior (e.g., *Mask Up, I do*; *I wear a mask*) vs. a collective behavior (e.g., *Mask Up, Together*; *We wear a mask*). The individualistic appeal also emphasized that wearing a mask is for individual considerations (e.g., *my life, my health*), whereas the collectivistic appeal highlighted that wearing a mask is due to collective or group considerations (e.g., *our loved ones, our community*). Additionally, the individualistic appeal included pictures featuring individual persons wearing masks, whereas the collectivistic appeal included pictures showing a group of people or family wearing masks.

To control for confounds, both versions of the poster used the same template, presenting the same layout and design. Texts followed the same script, with necessary variations for the experimental manipulation. The posters had almost the same word count. Readability indices of Flesch Reading Ease and Flesch-Kincaid Grade Level were approximately equal for the posters and suggested the texts on the posters are plain English and easy to understand for a 5th or 6th grader (Flesch, 1979).

A pretest of the stimuli was conducted with 131 undergraduate students at a U.S. university^1^. Participants were randomly assigned to view one of the posters, and then answered questions related to their perceptions of the message. Results showed that people (99.24%) could correctly identify the message frame to be individualistic or collectivistic. Also, people perceived the two posters to be equally comprehensible, *t* (129) = 1.08, *p* = 0.28, and have equal levels of visual attractiveness, *t* (129) = 0.40, *p* = 0.69. People found both posters pretty easy to read and understand (*M* = 6.31, *SD* = 0.86) and fairly attractive (*M* = 5.42, *SD* = 1.23) on 7-point scales.

### Measurement

Confirmatory Factor Analysis (CFA; Hunter and Gerbing, 1982) tested a unidimensional model for each scale. All scales showed acceptable internal validity and reliability. An average score for each scale was computed to represent each participant's standing on a certain variable. Measurements included in this study are described below.

Attitudes Toward Wearing Masks: Four 7-point semantic differential scale items measured the extent to which participants thought wearing masks outside their own houses during COVID-19 is positive, wise, desirable, and beneficial. CFA indicated acceptable model fit [χ^2^ (2) = 4.71, *p* = 0.10, *CFI* = 0.996, *RMSEA* = 0.057, *SRMR* = 0.015]. Cronbach's α = 0.85. This measure was administered prior to message exposure (*M* = 5.78, *SD* = 1.49) and after message exposure (*M* = 6.01, *SD* = 1.42). Higher scores on this scale meant more favorable attitudes toward wearing masks.

Behaviors/Behavioral Intention to Wear Masks: Eight 5-point Likert scale items formed an index for how much participants wear masks when they visit various public spaces including church/religious service, friend's/relative's house, gym/fitness studio, theater/museum, park/beach, on streets/roads, around my neighborhood/community block, and drive-thru service. These locations were selected because survey data (Key, 2021) showed that there existed reasonable variations in people's behaviors of wearing masks at these locations. In contrast, for places such as grocery stores, public transportation, or hospitals, where mask mandates were in effect at the time this study was conducted, people's behaviors lack variance (Key, 2021). Participants indicated their responses with 1 = *never*, 2 = *sometimes*, 3 = *about half the time*, 4 = *most of the time*, 5 = *always*, or NA = *I have not been to/do not plan to go to this place*. An average score was calculated for each participant. Higher scores on this index meant more behaviors/behavioral intention to wear masks. This measure was administered prior to message exposure (*M* = 3.57, *SD* = 1.32) and after message exposure (*M* = 3.90, *SD* = 1.29). Prior to message exposure, about half of the participants (53.2%) in this study reported that they wore masks most of the time or always when they visited public spaces, with more Asian Americans (64.6%) than European Americans (40.8%) consistently doing so. The baseline levels suggested reasonable room for changes to occur, so ceiling effect was not a severe concern.

Collectivism: Four 7-point Likert scale (1 = *strongly disagree* to 7 = *strongly agree*) items sampled from Boykin et al. (1997) measured the extent to which participants valued social ties, interdependence, group responsibility, and group welfare. CFA indicated acceptable model fit [χ^2^ (2) = 0.51, *p* = 0.77, *CFI* = 1.00, *RMSEA* = 0.00, *SRMR* = 0.006]. Cronbach's α = 0.77. Higher scores on this scale meant higher levels of collectivism. An independent sample *t*-test showed that European Americans (*M* = 4.71, *SD* = 1.00) scored significantly lower on collectivism than Asian Americans (*M* = 5.43, *SD* = 0.94) in this study, *t* (416) = 7.54, *p* < 0.001.

Message Engagement: Four 7-point semantic differential scale items adapted from Lee and Aaker (2004) measured the extent to which participants were involved, focused, paid attention, and read the message carefully. The wording of items was revised for the context of this study. CFA indicated acceptable model fit [χ^2^ (2) = 9.14, *p* = 0.01, *CFI* = 0.984, *RMSEA* = 0.092, *SRMR* = 0.028]. Cronbach's α = 0.75. Higher scores on this scale meant higher levels of message engagement (*M* = 5.80, *SD* = 1.18).

Perceived Message Relevance: Four 7-point Likert scale (1 = *strongly disagree* to 7 = *strongly agree*) items adapted from Jensen et al. (2012) measured the extent to which participants perceived the message to be relevant, important, and applicable to themselves. The wording of items was revised for the context of this study. CFA indicated acceptable model fit [χ^2^ (2) = 3.23, *p* = 0.20, *CFI* = 0.999, *RMSEA* = 0.038, *SRMR* = 0.004]. Cronbach's α = 0.96. Higher scores on this scale meant higher levels of perceived message relevance (*M* = 5.77, *SD* = 1.34).

Counter-arguing: Three 7-point Likert scale (1 = *not at all* to 7 = *very much so*) items from Silvia (2006) measured the extent to which participants criticized the message, thought of points that went against the message, and were skeptical of the message while viewing the message. Cronbach's α = 0.89. Higher scores on this scale meant higher levels of counter-arguing while viewing the message (*M* = 2.71, *SD* = 1.81).

## Results

### The effects of individualistic vs. collectivistic appeals

**Figure 1:**
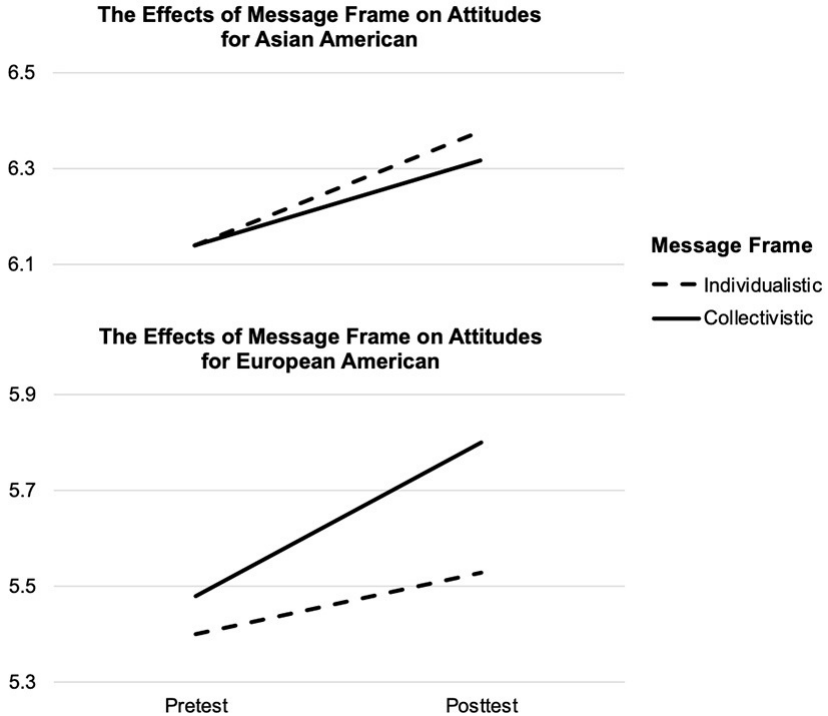
The effects of message frame on attitudes by ethnicity.

Data were analyzed using *SPSS* (RRID:SCR_016479). Listwise deletion was applied in case of missing values. To examine the effects of individualistic vs. collectivistic appeal on attitudes toward wearing masks, a repeated-measures analysis of variance was conducted, with message frame and audience ethnicity as between-subjects factors (see Figure 1). Tests of between-subjects effects showed that overall Asian Americans had more favorable attitudes toward wearing masks than European Americans, *F*_(1, 414)_ = 28.62, *p* < 0.001, η^2^ = 0.06. Tests of within-subjects effects showed that overall participants reported more favorable attitudes toward wearing masks after message exposure, *F*_(1, 414)_ = 21.55, *p* < 0.001, η^2^ = 0.05.

Although the interaction effects were not statistically significant, Figure 1 suggested that there were more nuances underlying the big picture. To take a closer look at the effects of individualistic vs. collectivistic appeal on attitude change by audience ethnicity, post-hoc paired sample *t*-tests were conducted. Mean scores by conditions were presented in Table 1. For European Americans, when exposed to an individualistic appeal, participants' attitudes at pretest (*M* = 5.40, *SD* = 1.61) and posttest (*M* = 5.53, *SD* = 1.55) did not differ significantly, *t* (96) = 1.49, *p* = 0.14. However, a collectivistic appeal caused a significant change in attitudes from pretest (*M* = 5.48, *SD* = 1.68) to posttest (*M* = 5.80, *SD* = 1.59), *t* (107) = 3.29, *p* < 0.01 (*p* = 0.001), Cohen's *d* = 0.32. Therefore, a collectivistic rather than an individualistic appeal effectively improved attitudes toward wearing masks for European Americans.

**Table 1:** Attitudinal outcomes by message frame and audience ethnicity.

		**Individualistic**	**Collectivistic**
European American	Pretest	5.40 (1.61)	5.48 (1.68)
	Posttest	5.53 (1.55)	5.80 (1.59)
Asian American	Pretest	6.14 (1.05)	6.14 (1.37)
	Posttest	6.38 (1.08)	6.32 (1.17)

N = 418. Standard deviations are in the parentheses.

As for Asian Americans, viewing an individualistic appeal resulted in significant attitude change, *t* (107) = 2.44, *p* < 0.05 (*p* = 0.02), Cohen's *d* = 0.24, from pretest (*M* = 6.14, *SD* = 1.05) to posttest (*M* = 6.38, *SD* = 1.08). Additionally, a collectivistic message frame was also effective in significantly changing attitudes among Asian Americans, *t* (104) = 2.03, *p* < 0.05 (*p* = 0.04), Cohen's *d* = 0.20, from pretest (*M* = 6.14, *SD* = 1.37) to posttest (*M* = 6.32, *SD* = 1.17). Furthermore, the effects of an individualistic and a collectivistic appeal on attitude change did not differ significantly, *F*_(1, 211)_ = 0.20, *p* = 0.66. Therefore, both an individualistic and a collectivistic appeal significantly improved attitudes toward wearing masks among Asian Americans.

**Figure 2:**
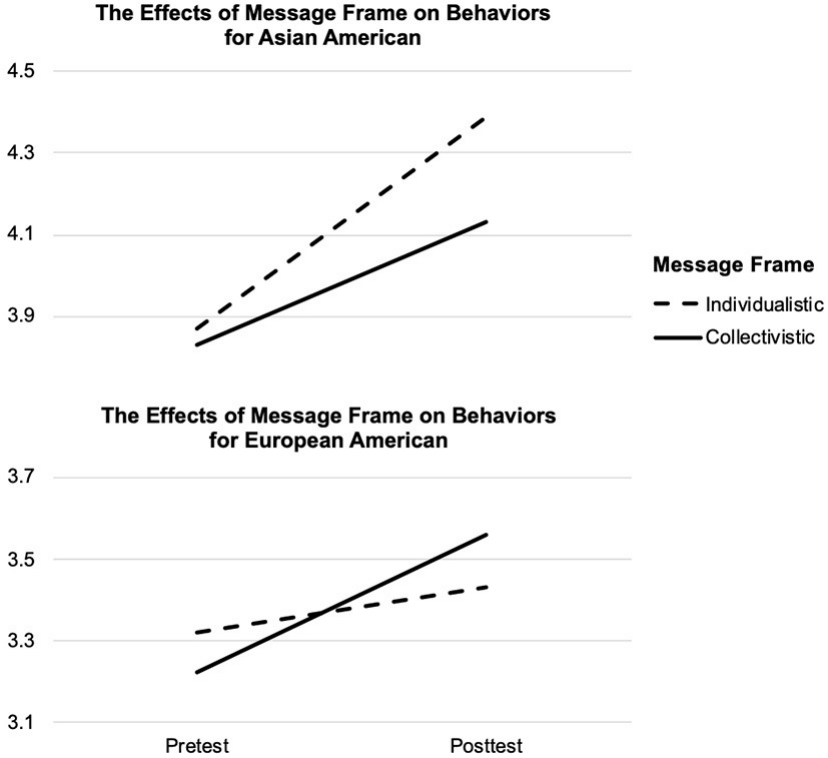
The effects of message frame on behaviors by ethnicity.

To examine the effects of message frame on behavioral intention to wear masks, a repeated-measures ANOVA was conducted, with message frame and audience ethnicity as between-subjects factors (see Figure 2). Tests of between-subjects effects showed that overall Asian Americans had higher behavioral intention to wear masks than European Americans, *F*_(1, 398)_ = 31.31, *p* < 0.001, η^2^ = 0.07. Tests of within-subjects effects showed that overall participants' behavioral intention to wear masks increased after message exposure, *F*_(1, 398)_ = 70.00, *p* < 0.001, η^2^ = 0.15. Additionally, the observed behavioral change was different depending on ethnicity, *F*_(1, 398)_ = 6.08, *p* < 0.05 (*p* = 0.01), η^2^ = 0.01, and ethnicity by message frame, *F*_(1, 398)_ = 9.14, *p* < 0.01 (*p* = 0.003), η^2^ = 0.02.

To further probe the effects of message frames on behavioral intention by ethnicity, paired sample *t*-tests were conducted. Mean scores by conditions were presented in Table 2. For European Americans, an individualistic appeal did not result in significant behavioral change from pretest (*M* = 3.32, *SD* = 1.41) to posttest (*M* = 3.43, *SD* = 1.31), *t* (88) = 1.40, *p* = 0.17. However, when European Americans were exposed to a collectivistic appeal, they reported significantly higher behavioral intention to wear masks at posttest (*M* = 3.56, *SD* = 1.40) compared to pretest (*M* = 3.22, *SD* = 1.41), *t* (102) = 4.31, *p* < 0.001, Cohen's *d* = 0.42. Therefore, a collectivistic rather than an individualistic appeal effectively increased behavioral intention to wear masks among European Americans.

**Table 2:** Behavioral outcomes by message frame and audience ethnicity.

		**Individualistic**	**Collectivistic**
European American	Pretest	3.32 (1.41)	3.22 (1.41)
	Posttest	3.43 (1.31)	3.56 (1.40)
Asian American	Pretest	3.87 (1.09)	3.83 (1.25)
	Posttest	4.39 (1.04)	4.13 (1.17)

N = 402. Standard deviations are in the parentheses.

When Asian Americans were exposed to an individualistic appeal, their behavioral intention increased significantly from pretest (*M* = 3.87, *SD* = 1.09) to posttest (*M* = 4.39, *SD* = 1.04), *t* (105) = 6.88, *p* < 0.001, Cohen's *d* = 0.67. Furthermore, a collectivistic appeal was also able to cause significant behavioral change among Asian Americans, *t* (103) = 4.21, *p* < 0.001, Cohen's *d* = 0.41, from pretest (*M* = 3.83, *SD* = 1.25) to posttest (*M* = 4.13, *SD* = 1.17). Therefore, both an individualistic and a collectivistic appeal significantly increased behavioral intention to wear masks among Asian Americans.

### Potential mechanisms for the effects of individualistic vs. collectivistic appeals

To investigate the mechanisms through which individualistic vs. collectivistic appeal had effects on attitudes toward wearing masks, mediation analyses (Hayes, 2009) were conducted with *PROCESS* (RRID:SCR_021369). List wise deletion was applied in case of missing values. Message frame was the independent variable, and it was dummy coded with the individualistic appeal as the reference condition. Posttest measure of attitudes was treated as the dependent variable, and pretest measure of attitudes was included as the covariate variable. Message engagement, perceived message relevance, and counter-arguing were tested as parallel mediating variables.

Results showed that there were no significant indirect effects of message frame on attitudes through any of the mediating variables. All the indirect effects had bootstrapped 95% confidence intervals including zero. Therefore, none of the proposed mediators were able to explain the effects of message frame on attitudes.

Similarly, mediation analyses were conducted to examine the mediating relationships between message frame and behavioral intention to wear masks. The mediation models included dummy coded message frame as the independent variable, posttest measure of behavioral intention as the dependent variable, pretest measure of behaviors as the covariate variable. The same set of mediating variables were tested.

**Figure 3:**
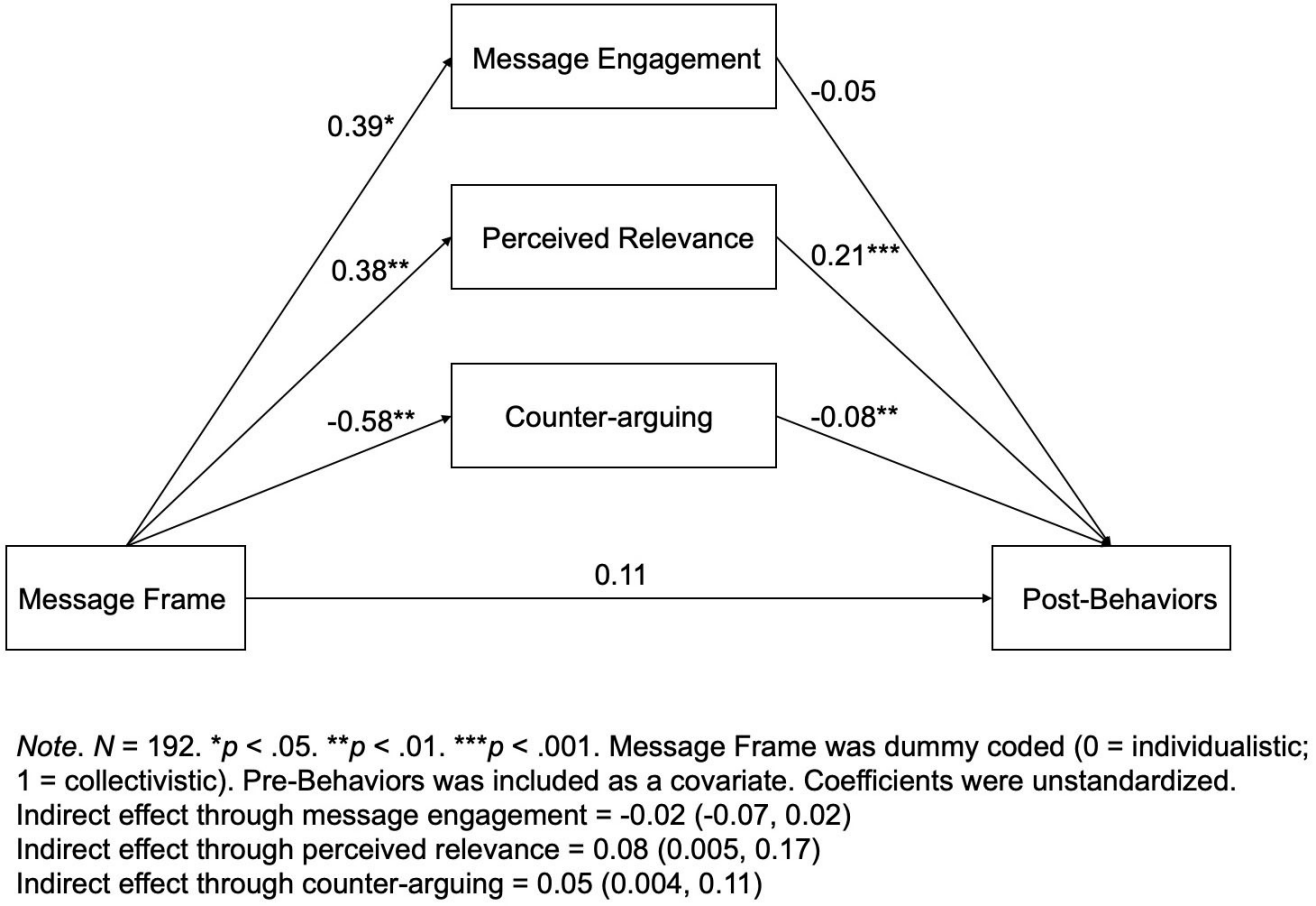
Parallel mediation model.

Results (see Figure 3) showed that for European Americans, perceived message relevance was a significant mediator. The indirect effect of message frame on behavioral intention to wear masks through perceived message relevance was 0.08, with a bootstrapped 95% confidence interval [0.005, 0.17]. European Americans thought a collectivistic appeal was more relevant than an individualistic appeal, which in turn led to increased behavioral intention. Additionally, counter-arguing also significantly mediated the relationship between message frame and behavioral intention among European Americans. The indirect effect through counter-arguing was 0.05, with a bootstrapped 95% confidence interval [0.004, 0.11]. European Americans engaged in less counter-arguing when seeing a collectivistic vs. an individualistic appeal, which in turn led to increased behavioral intention. However, message engagement was not a significant mediator between message frame and behavioral intention, as a bootstrapped 95% confidence interval around the indirect effect included zero. Therefore, perceptions of message relevance and levels of counter-arguing were able to explain the differential effects of individualistic vs. collectivistic appeal on behavioral intention to wear masks for European Americans.

## Discussion

This study investigated the persuasive effects of individualistic and collectivistic appeal among European Americans and Asian Americans and explored potential mechanisms through which cultural value appeals had impacts on attitudinal and behavioral outcomes. Results showed that for European Americans, a collectivistic but not an individualistic appeal was effective in improving attitudes and behavioral intention. For Asian Americans, both individualistic and collectivistic message frames predicted improved attitudes and behavioral intention. Perceived message relevance and counter-arguing were significant mediators explaining the effects of collectivistic (vs. individualistic) appeal on behavioral outcomes for European Americans. These findings had important theoretical and practical implications.

To begin with, compared to a collectivistic appeal, an individualistic appeal evoked more counter-arguing, which in turn led to less behavioral intention among European Americans. It seemed that European Americans in this study experienced stronger psychological reactance when they saw an individualistic appeal. This might partly be that when their values of individualism were employed to persuade them, they experienced psychological discomfort and dissonance (Festinger, 1957). They might saw an individualistic appeal as a challenge or a threat to their values and identities. To resolve these negative feelings, they engaged in defensive processing of the message to minimize the message (Ko and Kim, 2010; Yang and Nan, 2019). As a result, an individualistic appeal failed to persuade European Americans in this study.

However, given that a collectivistic appeal was effective with Asian Americans, one might question why Asian Americans in this study did not see a collectivistic appeal as threatening to their values and engaged in counter-arguing, like European Americans did with an individualistic appeal. One of the reasons might be that Asian Americans had a relatively high baseline level of the recommended behavior, compared to European Americans in this study. The position the messages advocated for might fall in Asian Americans' latitude of acceptance or non-commitment but in European Americans' latitude of rejection (see Social Judgment Theory, Sherif et al., 1965). It is possible that psychological reactance was triggered when recipients found the message's position unreasonable or objectionable, but not when they thought it was somewhat acceptable or when they were non-committed. For Asian Americans, a collectivistic appeal was value-matching and was perceived to be more related or applicable to them and their situation and providing culturally congruent views of and solutions to the issue at hand (Kreuter and Wray, 2003; Rimer and Kreuter, 2006; Hawkins et al., 2008; Jensen et al., 2012).

Moreover, this study found that an individualistic appeal was as effective as a collectivistic appeal with Asian Americans. It seemed to suggest that Asian Americans in this study found an individualistic view of the issue to be congruent with their values as well. One possible explanation might be related to the concept of collectivism. A key element of collectivism is contextual self (Markus and Kitayama, 1991; Triandis, 1995). People from collectivistic cultures may change their views of oneself according to the context or situation— “how I behave depends on who I am with, where I am, or both” (Oyserman et al., 2002, p. 9). An individualistic or a collectivistic appeal may be a form of situational priming (Aaker and Lee, 2001) for Asian Americans in this study, so both individualistic and collectivistic appeals were compatible with them. Another possible explanation might be related to the acculturation of Asian Americans. Acculturation is a process of adaptation to the cultural values, attitudes, and practices of the dominant or prevalent culture (Berry, 1997, 2005). In the United States, the prevalent culture is largely European based. Although Asian Americans in this study reported to be more collectivistic, they may have also acquired individualistic cultural characteristics over time (Zhou, 2004). Some scholars argue that individualism and collectivism are not opposites on a continuum, but rather two orthogonal concepts (Oyserman et al., 2002). It is possible that Asian Americans are high on both collectivism and individualism as they develop a bicultural identity (Berry et al., 1987) through acculturation processes. If so, both individualistic and collectivistic appeals might be perceived as culturally congruent by Asian Americans, and thus both were effective.

Furthermore, a collectivistic appeal was effective for both European and Asian Americans in this study. Aside from the discussions above, another factor may be related to the health context of this study. To prevent the spread of an infectious disease asks for collective efforts and for the society/community to take actions together. Measures to reduce disease transmission, such as social distancing, getting vaccination, and wearing masks, not only benefits the individual, but also simultaneously protects others. The extent to which this is the case, people might perceive a collectivistic appeal to be very congruent with the health issue, and thus thought a collectivistic appeal to be very relevant and applicable in processing the message. Several studies somewhat supported this idea, such as Yu and Shen (2013) on getting a flu shot, Ko and Kim (2010) on safe sexual practice and condom use, and Courtney et al. (2022) on COVID-19 prevention. In contrast, for non-infectious conditions, such as to reduce caffeine consumption (Uskul and Oyserman, 2010) or cancer screening (Han and Jo, 2012), individuals can take responsibility for one's own health. And yes, one's own health can have relational consequences (e.g., your loved ones will hurt to see you ill). In this case, both individualistic and collectivistic appeals could apply, and message effectiveness could depend on how much one values individualism—collectivism.

Admittedly, this study has some limitations and refers to future directions of investigation. Firstly, the assessment of participants' cultural value orientation was not optimal, since there may be more nuances in cultural value dimensions as discussed above. On top of that, Asian Americans oftentimes link their identities to specific countries of origin and there are generational differences in terms of how Asian Americans perceive their identities (Zhou, 2004). Future studies may apply better and more delicate assessments of cultural values. Additionally, this study did not include a manipulation check of message frame, since “when message variations are defined in terms of intrinsic features, message manipulation checks are unnecessary” (O'Keefe, 2003, p. 251, 252). However, it may be useful to assess participants' perception of the message frame manipulation in the main study, even though the manipulation has been pretested to work as intended. Furthermore, findings based on one specific health context may not be generalizable to other contexts where the key determinants of health outcomes may be different. Future studies are encouraged to continue testing the effects of cultural value appeals in other contexts. As a final thought, ethnic minority groups in the U.S. have long been disproportionally affected by various health issues and experiencing health inequity. Cultural value appeals as a form of cultural-sensitive communication can potentially help reduce health disparities to the extent that it makes health messages equally understandable, meaningful, and effective to people from various ethnic cultural backgrounds (Betsch et al., 2016).

## Data availability statement

The raw data supporting the conclusions of this article will be made available by the authors, without undue reservation.

## Ethics statement

The studies involving human participants were reviewed and approved by the Institutional Review Board at Michigan State University. The patients/participants provided their written informed consent to participate in this study.

## Author contributions

The author confirms being the sole contributor of this work and has approved it for publication.

## Funding

This study was funded by the Graduate School and the Health and Risk Communication Center at Michigan State University. Support was also provided by the Young Scholar Research Start-up Fund (10822445) at China University of Political Science and Law.

## Conflict of interest

The author declares that the research was conducted in the absence of any commercial or financial relationships that could be construed as a potential conflict of interest.

## Publisher's note

All claims expressed in this article are solely those of the authors and do not necessarily represent those of their affiliated organizations, or those of the publisher, the editors and the reviewers. Any product that may be evaluated in this article, or claim that may be made by its manufacturer, is not guaranteed or endorsed by the publisher.

## Footnotes

^1^Participants were 19.89 years old on average (*SD* = 2.19). 63.4% of the participants were female, and 36.6% were male. Participants self-identified their ethnicity to be White (69.5%), Black (10.7%), Asian (9.9%), Hispanic (4.6%), or other (5.3%). Participants' political orientation score was 4.28 (*SD* = 1.39) on a 7-point scale ranging from very conservative to very liberal.
