# Supplementary material for: Being targeted: How counter-arguing and message relevance mediate the effects of cultural value appeals on disease prevention attitudes and behaviors
Source: Front Psychol. 2022 Sep 29;13:1018402. doi: 10.3389/fpsyg.2022.1018402 (PMC9558217; doi:10.3389/fpsyg.2022.1018402)
Supplement: Supplementary file 1 [file Table_1.DOCX]

Supplementary Material


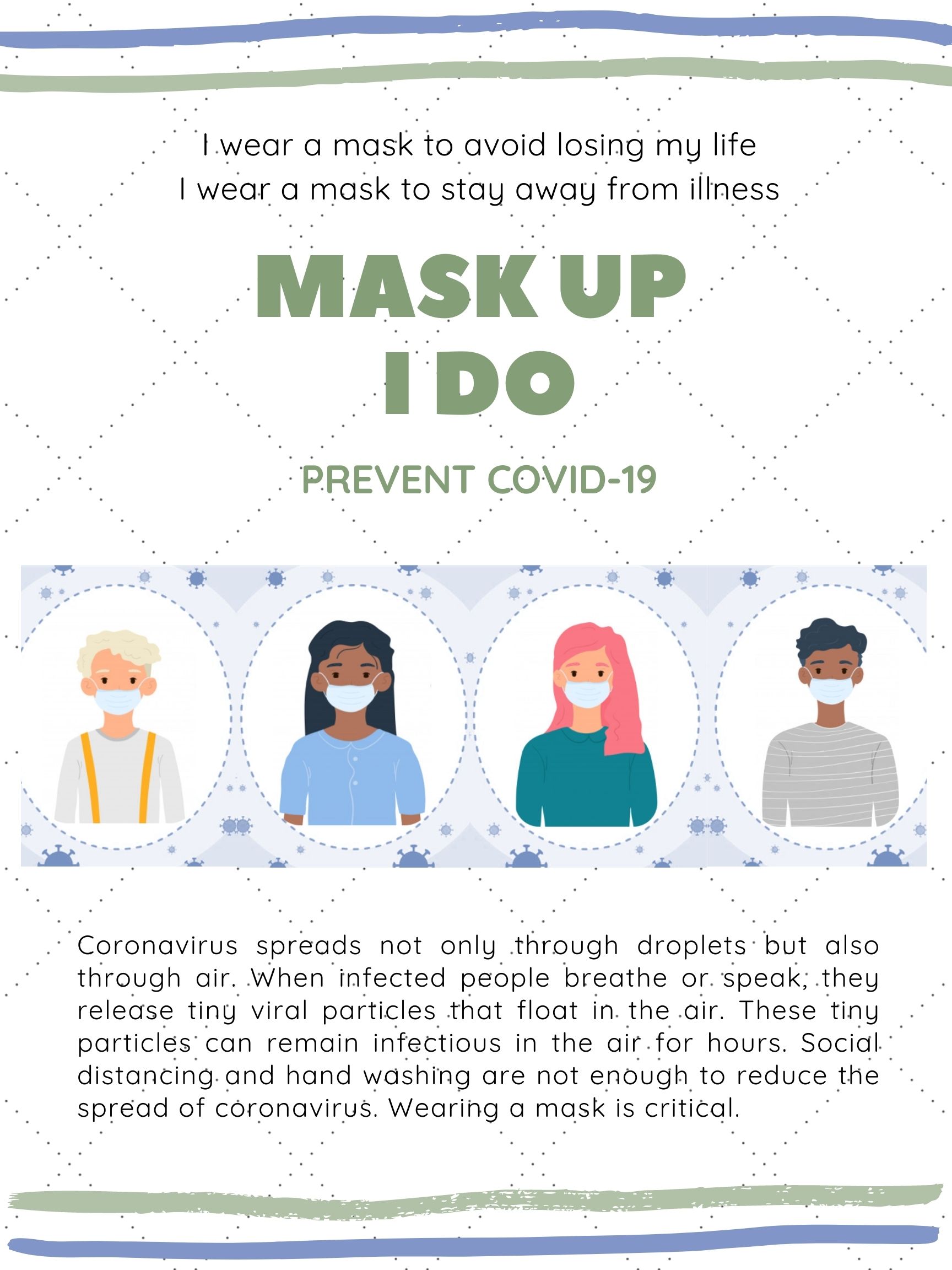


Supplementary Image 1. Experimental stimuli (individualistic appeal)


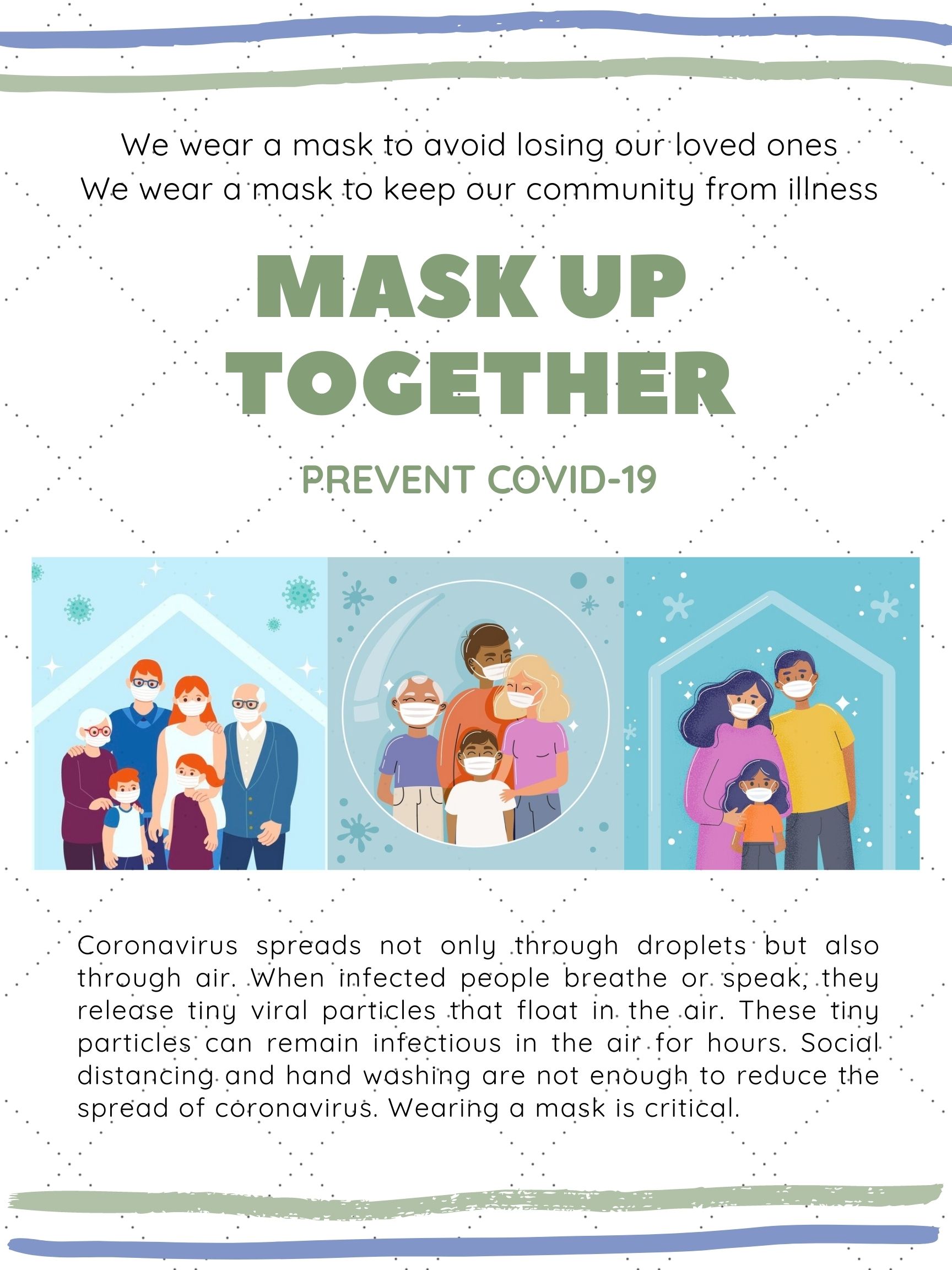


**Supplementary Image 2.** Experimental stimuli (collectivistic appeal)
